# Cesarean scar pregnancy with devastating profuse vaginal bleeding

**DOI:** 10.1093/jscr/rjab566

**Published:** 2022-01-15

**Authors:** Brahmana Askandar Tjokroprawiro, Muhammad Ilham Aldika Akbar

**Affiliations:** Departement Obstetrics Gynecology, Dr. Soetomo General Academic Hospital, Faculty of Medicine – Universitas Airlangga, Surabaya, Indonesia; Departement Obstetrics Gynecology, Universitas Airlangga Hospital, Faculty of Medicine – Universitas Airlangga, Surabaya, Indonesia

## Abstract

A cesarean scar pregnancy (CSP) is a rare type of ectopic pregnancy that does not have any obvious signs or symptoms. However, the gestational sac in CSP is often embedded in the myometrial scar from the previous cesarean section. We report two cases of CSP in women with a history of cesarean sections who experienced profuse vaginal bleeding. The patients underwent hysterectomy at their own request due to devastating bleeding. CSP is one of the complications of cesarean sections. The patient may present with devastating bleeding, and immediate management is necessary. In a woman who is early into her pregnancy and has a history of cesarean section with profuse vaginal bleeding, CSP is one of the possible diagnoses.

## INTRODUCTION

Cesarean scar pregnancy (CSP) is described as the placement of a gestational sac within the scar from a previous cesarean operation and is considered an ectopic pregnancy. It can have major effects such as devastating bleeding and uterine rupture. CSP occurs in 1 in 1800–2200 pregnancies and accounts for 6% of all ectopic pregnancies in women who have previously had a cesarean delivery [1]. CSP has no pathognomonic signs or symptoms, and its presentation varies considerably. We report two cases of CSP with devastating vaginal bleeding without intraabdominal bleeding in women during early pregnancy.

## CASE 1

**Figure 1:**
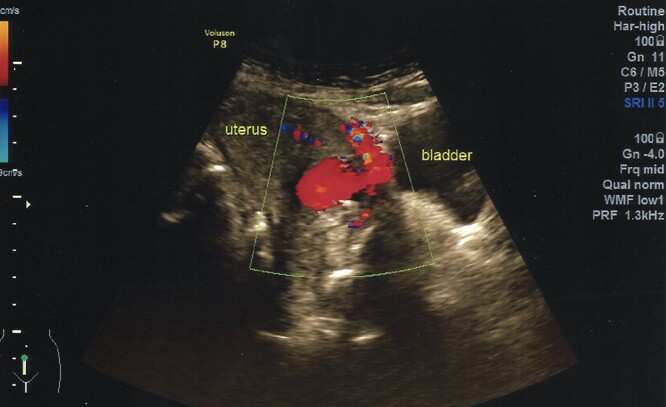
Ultrasonography demonstrates a rich blood flow in the lower uterine segment with a normal fundus.

**Figure 2:**
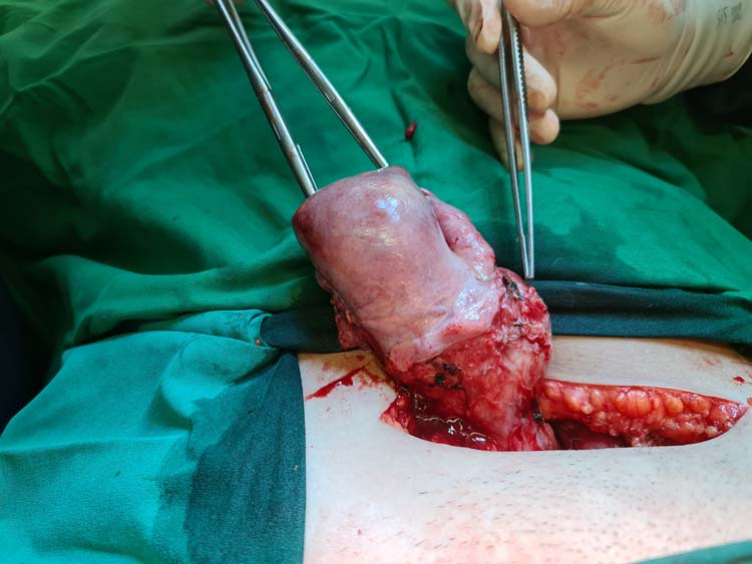
A bulging mass with intact serosa was observed in the left lower uterine segment.

**Figure 3:**
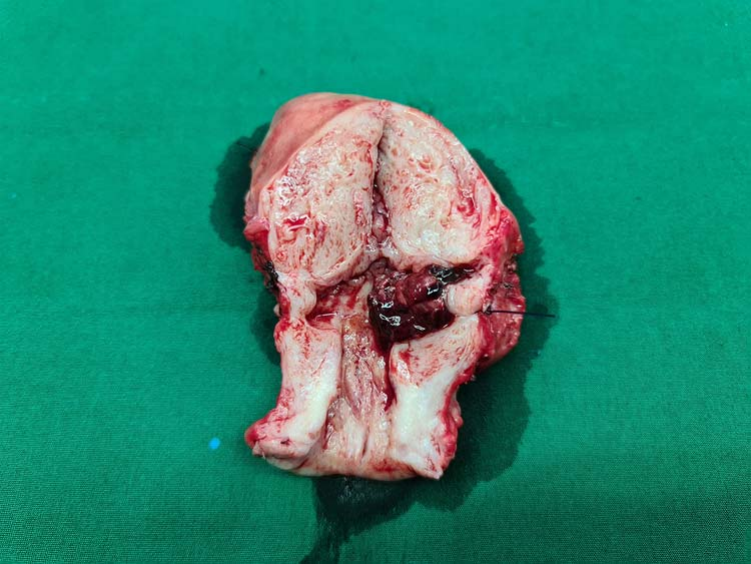
The gross specimen showing a cesarean scar pregnancy.

**Figure 4:**
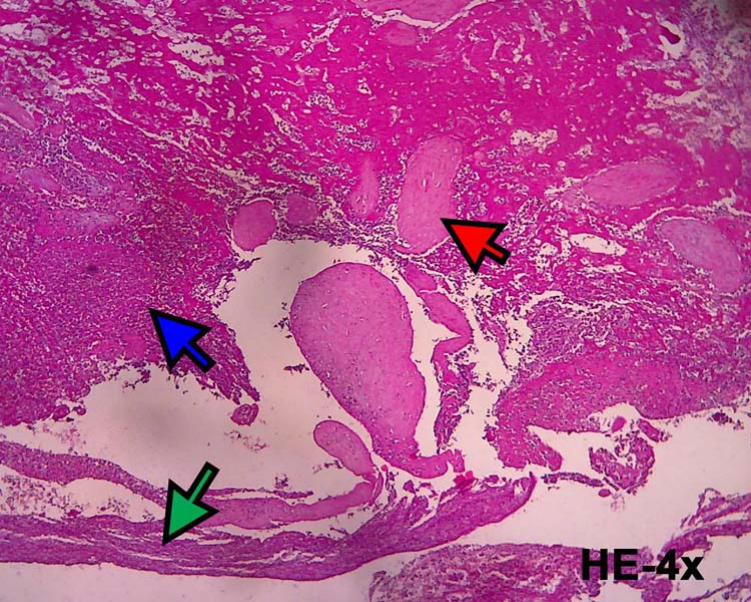
Histopathological examination confirmed the diagnosis of cesarean scar pregnancy (red arrow = necrotic villi chorealis; green arrow = fibromuscular tissue and blue arrow = necrotic tissue).

A 40-year-old woman came to the emergency department of the tertiary hospital with vaginal bleeding. She had a history of two cesarean sections (CS), and the last one was 3 years prior without any contraception. She was 10 weeks pregnant and had gone through abnormal uterine bleeding for 2 months. The patient arrived at the emergency department conscious with a blood pressure of 80/40, heart rate of 112 beats/min and hemoglobin level of 11.2 g/dl. Two bags of Ringer’s lactate solution (1000 ml) were given to stabilize the patient. After the patient was stable, a vaginal examination was performed. The cervix was normal, and a small amount of bleeding came out from the external os of the cervix. Ultrasound examination was performed. It showed blood flow in the lower anterior segment of the uterus, but the gestational sac could not be seen, and no intraabdominal bleeding was observed (Fig. 1). However, the pregnancy test pack showed a positive result. The patient underwent observation in the emergency department. At 2 h after admission, the patient underwent profuse vaginal bleeding. The blood pressure was 70/40 with a heart rate of 122 beats/min and decreased hemoglobin level of 6.5 g/dl. The patient consented to undergoing hysterectomy. Laparotomy was performed after hemodynamic stabilization. Intraoperative exploration showed no intraabdominal bleeding. The size of the uterus was normal, no uterine perforation was observed, both adnexa were normal and a bulging mass in the left lower uterine segment was observed without any uterine perforations (Fig. 2). Hysterectomy was performed. The gross specimen indicated a CSP (Fig. 3), which was confirmed by histopathological examination (Fig. 4). The patient received blood transfusion intra- and postoperatively. On Day 3 after surgery, the patient was in good, stable condition and was discharged from the hospital.

## CASE 2

A 40-year-old woman who was 16 weeks pregnant and had vaginal bleeding came to the hospital. Her blood pressure was 110/65, and the hemoglobin level was at 11.8 g/dl. No abdominal pain was observed. Her first child was delivered via CS 3 years before the current pregnancy. Ultrasonography showed a heterogenous mass suspected to be blood clots in the lower segment of the uterus. No intraabdominal bleeding was observed. The physical examination result was normal. Vaginal examination showed bleeding was coming from the uterus, but no tissue could be observed. The patient underwent observation in the emergency room. Two hours after admission, the patient experienced profuse vaginal bleeding. The blood pressure dropped to 60/35. Crystalloid fluid was given to maintain normovolemia during the course of the bleeding. The hemoglobin level dropped to 5.3 g/dl. We discussed with the patient and her family the need for immediate surgery to stop the bleeding. The patient requested that her uterus be removed if necessary to save her life. The patient was taken to the operating room after written consent was obtained. Blood for transfusion was ordered and given during surgery.

**Figure 5:**
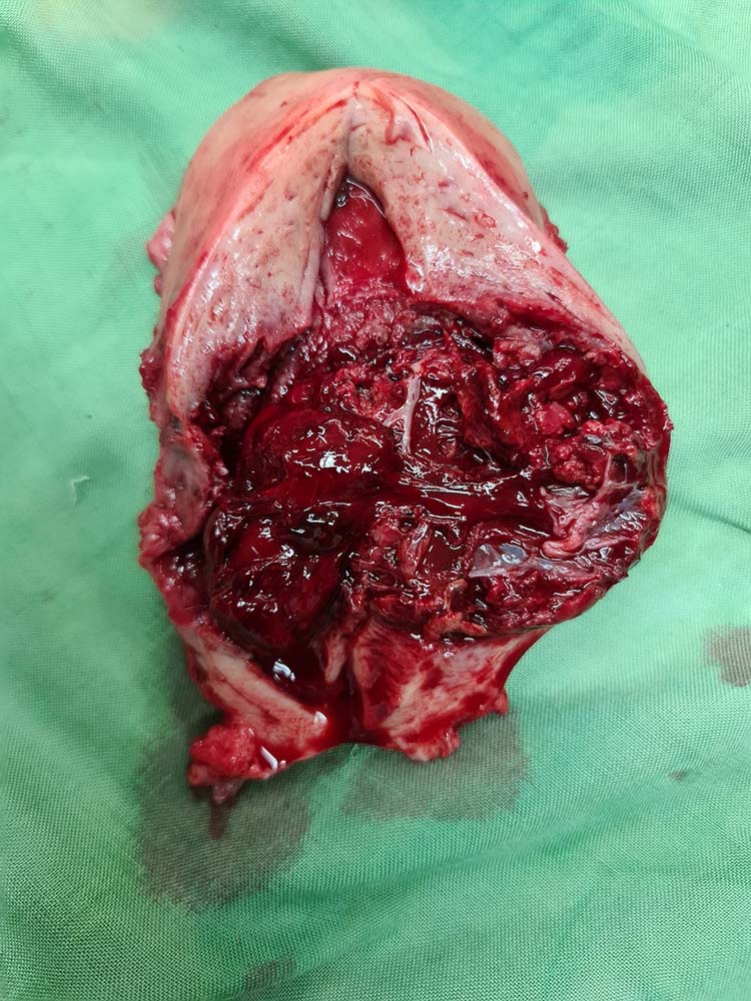
Gross specimen of the uterus. A mass in the lower uterine segment was observed, which did not penetrate the serosal layer.

**Figure 6:**
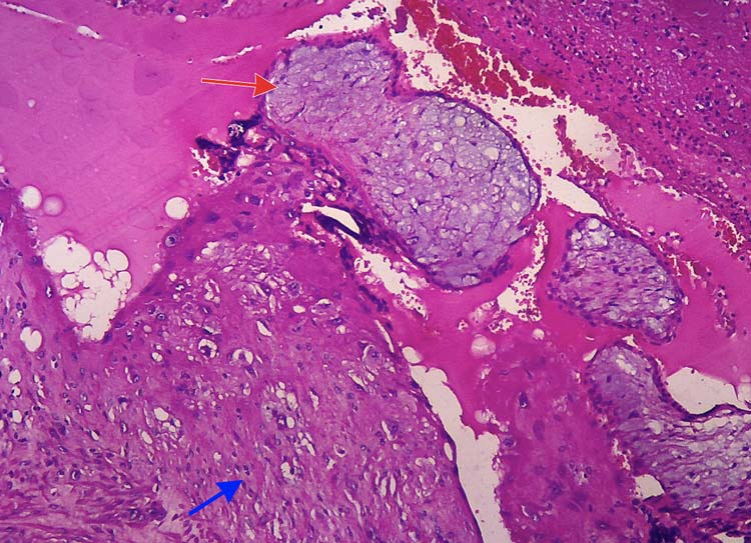
Microscopic image (hematoxylin and eosin, 40×) showing villi chorealis embedded in fibromuscular tissue (red arrow: villi chorealis and blue arrow: fibromuscular tissue).

Laparotomy was performed. A midline skin incision was made to remove the midline skin incision scar from the previous CS. The lower segment of the uterus was enlarged (bulging) with the serosa intact. No intraabdominal bleeding was observed. Both adnexa were normal. Total hysterectomy was performed. The gross specimen showed a bulging mass with intact serosa in the lower segment of the uterus (Fig. 5). The operative time was 30 min with a surgical blood loss of 50 ml. Three bags of packed red cells and two bags of whole blood were transfused. One day after surgery, the hemoglobin level increased to 8.2 g/dl, and the patient’s condition stabilized considerably. The patient was discharged from the hospital on the third day after surgery. Histopathological results confirmed the diagnosis of CSP with a size of 6 cm × 6 cm (Fig. 6).

## DISCUSSION

Larsen and Solomon reported the first incidence of CSP in 1978 [2]. It was an early pregnancy following uterine curettage due to persistent heavy bleeding and acute abdominal pain. During laparotomy, the presence of fetal tissues was observed in the recess of the CS scar.

CSP is a type of ectopic pregnancy in which the gestational sac is embedded in the myometrium and scar from a previous CS. Between 1990 and 2018, the average global CS rate climbed by 19 percentage points. The highest rise occurred in less developed countries, whereas the smallest gain occurred in least developed countries [3]. The incidence of CSP has also risen with CS rate. One institution showed an increase in CSP incidence from 1 case in 2013 to 12 cases in 2016 [1].

CSP is extremely uncommon and occurs in 1 in every 1800–2216 pregnancies [4]. The mean age of patients with CSP was 33.4 ± 5.7 years and was unaffected by the number of previous cesarean deliveries [4]. The patients in the present case study demonstrated histories of one and two cesarean deliveries. The period between the last cesarean delivery and the occurrence of CSP varied between 6 months and 12 years. The cases in the present study showed a 3-year interval. A study of 64 cases showed that the mean gestational age at the time of diagnosis was 6.5 ± 1.1 weeks [5]. In our study, the gestational ages were 8 and 16 weeks. The mean number of previous CS was 1.6 ± 0.6 [5].

Transabdominal ultrasonography was performed in both patients, which showed a bulging mass in the lower segment of the uterus with a prominent vascularization in both cases. No signs of live intrauterine pregnancy were detected. The gestational sac could not be identified as the image was dominated by blood clots. The fundus of the uterus in both cases was normal without any adnexal mass detected. Several criteria can be used to diagnose CSP in the first trimester by transvaginal sonography: empty uterus, empty cervical canal, gestational sac embedded in the anterior lower uterine segment; and thin or absent myometrium between the sac and the bladder [6]. The main findings from ultrasonography are the implantation of the gestational sac in the previous cesarean scar and the thinning of the anterior myometrium at an average of 3.0 ± 2.0 mm [5]. In patients with excessive vaginal bleeding during early pregnancy, the imaging options are extremely limited. Signs that can be used as a guide to direct the diagnosis of CSP are an early pregnancy with complaints of profuse vaginal bleeding and ultrasonography showing a mass in the lower anterior uterine segment and no intraabdominal bleeding.

The management of CSP varies between surgical and nonsurgical management. The nonsurgical management is uterine artery embolization (UAE) plus local or systemic methotrexate [7–9]. A study on 60 cases of CSP showed that expectant management is associated with a high risk of hysterectomy because of morbidity of the adherent placenta [10]. Another study showed that the success rate of systemic methotrexate was 8.7%, whereas the success rate of UAE was 18.3% [11]. Surgical techniques with direct visibility appear to be safer and more effective than medicinal treatment with methotrexate alone [5]. In cases where the condition of the patient is hemodynamically unstable, the options for treatment are limited. Conservative surgery for a large CSP is difficult, such as in the second case in this study. The only definitive treatment is hysterectomy [12]. However, no widespread agreement on the optimal management strategy has been established. Numerous case reports and case series have documented successful outcomes when medical treatment, surgery, interventional radiology or a combination of these methods is used. Evidence-based management is not clear, and individualized treatment is the best option. Surgical management has the best success rate (100%), followed by medical treatment (53%) and conservative treatment with dilatation and curettage (0%; [13]). Conservative treatment has a risk of recurrence in the subsequent pregnancy.

If a patient in the early stages of pregnancy presents with profuse bleeding and a history of cesarean delivery, a diagnosis of CSP should be considered. Our case study shows that CSP can result in fatal profuse vaginal bleeding and demonstrates that cesarean delivery can result in complications; therefore, it should be performed only when medically indicated.
